# Usability Evaluation of a Smart Textile-Based Cushion System for Ubiquitous Breathing Exercises

**DOI:** 10.3390/s26154933

**Published:** 2026-08-04

**Authors:** Shonal Fernandes, Carolina García-Vázquez, Alberto Ramos, Negin Jahanbakhsh, Abdelakram Hafid, Mario Vega-Barbas

**Affiliations:** 1 Facultad de Diseño y Tecnología, University of Design, Innovation and Technology (UDIT), 28016 Madrid, Spain; carolina.garcia@udit.es (C.G.-V.); alberto.ramos@udit.es (A.R.); 2 ETSIS de Telecomunicación, Universidad Politécnica de Madrid, Calle Nikola Tesla S/N, 28038 Madrid, Spain; tonegin.jahanbakhsh@alumnos.upm.es (N.J.); mario.vega@upm.es (M.V.-B.); 3 Department of Computer Science & Engineering, Mälardalen University, 72220 Västerås, Sweden; abdelakram.hafid@mdu.se

**Keywords:** breathing exercises, monitoring, respiratory, smart textiles, self-intervention exercises, usability testing, ubiquitous computing, wearable

## Abstract

Smart textiles offer promising opportunities to address multiple aspects of relaxation-focused mental health interventions, which include monitoring and therapy. This paper presents a usability evaluation of a cushion-based smart textile system called Amiga, designed to guide users through breathing exercises that could be used in the future to support relaxation-focused interventions intended for independent use. A experimental user study with 20 participants was conducted to assess the system’s effectiveness, user performance, user experience, and overall satisfaction using structured interviews and validated usability questionnaires. The system achieved a mean System Usability Scale (SUS) score of 81.875 with standard deviation (σ = 11.8), exceeding the established SUS benchmark of 68 and corresponding to the ‘Excellent’ category rating, under a predefined assistance-free criterion. The system was designed for respiratory phases (Ph) and respiration rate (RR) monitoring through a guided-breathing exercise where 90% completed with only 40% passing the quality criteria, achieving an average pace synchronization accuracy (PSA) of 61.2 % and phase duration accuracy (PDA) of 30% with a tolerance of ±1 s, thus providing a initial baseline for autonomous use and highlighting opportunities to improve on-boarding and in-application guidance. Most participants reported positive feedback regarding their confidence and overall satisfaction. Usability challenges were identified related to physical interaction with the cushion and clarity of on-screen instructions, providing clear directions for future design refinement. These findings demonstrate the feasibility of smart textile-based devices for home-based breathing exercises.

## 1. Introduction

Breathing techniques have been proposed as a first-line and supplementary treatment for stress and anxiety [1]. Diaphragmatic breathing has demonstrated effectiveness in reducing stress, showing measurable improvement in the stress sub-scale of the Depression Anxiety Stress Scales—21 (DASS-21) [2]. Breathwork techniques serve as practical interventions for clinical mental health counsellors, integrating physiological approaches into treatment that traditionally require human-guided training, multiple sessions, and long-term practice [3,4]. However, access to such structured interventions remains limited for many individuals, highlighting the need for accessible, home-based alternatives.

The concept of ubiquitous computing opens new possibilities for mental health therapy [5], enabling the creation of platforms that enhance care by identifying people in need of treatment, accelerating access, and monitoring progress during or after intervention [6]. Ambient intelligent systems that continuously and naturally assess mental health and cognitive status through behavioral monitoring can facilitate therapeutic interventions for chronic conditions, reducing the burden on caregivers and healthcare institutions [7].

Smart wearable technologies have shown significant improvements in the monitoring of physiological signals, enabling the understanding of a person’s physical and mental state [8]. Although wearables can monitor and detect common mental health disorders such as stress and anxiety, they face important limitations in terms of comfort and usability. The main barrier to adoption is the general reluctance and discomfort experienced by users [9], with 32% of users abandoning wearable devices after six months and 50% after one year—a pattern particularly pronounced in mental health technology [10].

Smart textiles have emerged as a promising alternative, enabling the implementation of traditional monitoring systems with the added advantages of long-term comfort and reduced skin irritation [11]. However, for smart textiles to achieve widespread acceptance, they must meet a broad range of user requirements. The number of smart textile devices currently available to consumers remains limited, not only because of technological constraints but also because of usability challenges. Integrating end users into the development process is therefore essential to understand their reactions and performance [12].

User experience with smart textiles can vary significantly due to differences in body shapes, sizes, and sensor placement [13]. Assessing material comfort is critical given the dynamic contact between the smart textile and the human body [14]. Age and gender also play important roles in technology acceptance, as older adults may experience greater difficulty adopting new systems, whereas men tend to show more positive attitudes toward new technology [15]. Effective smart textile interfaces should appeal to both the visual and haptic senses, providing a seamless experience that does not require prior training [16].

Usability, defined as the extent to which a product enables users to achieve their goals effectively, efficiently, and with satisfaction [12], is a key concept and a mandatory requirement for successful product adoption. Usability evaluation integrates the user into the design process to ensure long-term acceptance and viability [12,17], and it plays a particularly important role in the early design stages by identifying and addressing interaction problems before deployment [18]. Despite increasing interest in smart textiles for health monitoring, relatively few studies have examined the usability of systems specifically intended to support everyday breathing exercises. This suggests that further work on user experience and acceptability in this context could be valuable.

This study is conducted to explore the usability and feasibility of the cushion-based smart textile system and its companion mobile application for wearable breathing exercise monitoring. Using the System Usability Scale (SUS) alongside structured interviews and direct observation to understand user experience and overall satisfaction and the breathing exercises to understand the feasibility of the system to determine the systems effectiveness and user performance this work aims to demonstrate how smart textiles could support accessible, affordable breathing exercises in a ubiquitous computing environment.

## 2. Materials and Methods

### 2.1. Description of the System

**Figure 1:**
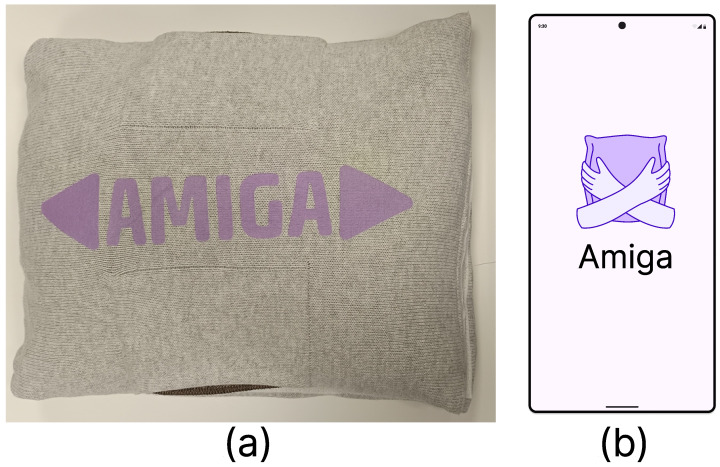
The Amiga system: (**a**) the cushion featuring the system name; (**b**) the mobile application initialization screen displaying the logo.

The system, named Amiga (Spanish for “female friend”), comprises a smart cushion and a companion mobile application, as illustrated in Figure 1. It was designed to support self-guided breathing exercises in a non-intrusive manner. The cushion integrates textile-embedded sensors while preserving user comfort during use. The mobile application communicates wirelessly with the cushion to record data, provide instructions, and guide users through guided breathing exercise.

The cushion was developed for respiratory phase detection and respiration rate monitoring. The respiration phase refers to the inhalation or exhalation process, and the respiration-rate refers to the number of respiration cycles (inhalation and exhalation) performed per minute. The main motivation of this study is to understand how participants would perform a guided breathing exercise where they would have to maintain the pace (20 Ph/min) and duration (3 s) of the phase in a controlled setting. The study was developed in such a way that participants were requested to follow the breathing instruction on the mobile application at the same time they held the cushion.

#### 2.1.1. The Smart Cushion

The core concept of the Amiga cushion is to provide a familiar and comforting object that people can hold during breathing exercises. The visual design adopts a calming color palette intended to enhance approachability for users engaging in relaxation exercises. The front surface of the cushion features the project logo “Amiga,” contributing to a positive and reassuring user experience and reinforcing the system’s identity.

**Figure 2:**
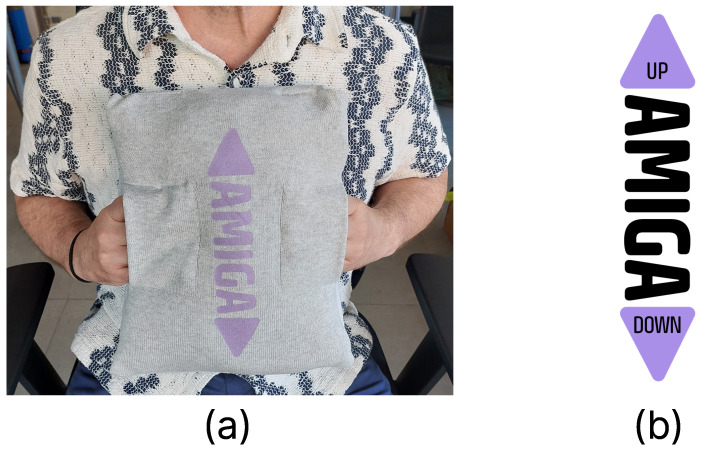
The Amiga cushion and design: (**a**) a person demonstrating the correct way to hold the cushion against the abdomen area; (**b**) the visual instruction printed on the back side of the cushion to guide participants with the orientation by which the cushion should be held.

To ensure reliable signal acquisition, participants are instructed to position the cushion on the chest, specifically centered near the xiphoid process, a key anatomical landmark located at the inferior end of the sternum and in proximity to the diaphragm. The recommended user positioning is illustrated in Figure 2a. Visual placement instructions are integrated on the reverse side of the cushion, as shown in Figure 2b, to guide correct orientation.

The system estimates the respiration rate, by phase detection, where inhalation and exhalation are detected using a textile-integrated sensing layer consisting of an embedded accelerometer that captures chest-wall motion associated with diaphragmatic activity. Data acquisition, preprocessing, and wireless transmission to a mobile application are managed by a low-power microcontroller via Bluetooth Low Energy (BLE).

A first-order finite difference is applied to the z-axis signal to estimate local slope variations. Positive and negative slopes correspond to inhalation and exhalation phases, respectively, enabling segmentation of the respiratory cycle and estimation of phase durations. RR is calculated by counting complete respiratory cycles over a 60 s interval. Communication between the sensing layer and application is asynchronous and event-driven via BLE.

#### 2.1.2. The Mobile Application

The mobile application serves as the primary user interface of the Amiga system. Developed for the Android ecosystem, it enables Bluetooth connectivity, data acquisition, guided breathing exercises, and real-time visualization of respiration rate. The application is not intended for clinical decision-making; its functionality is limited to data preprocessing, visualization, and user guidance during interaction with the system.

**Figure 3:**
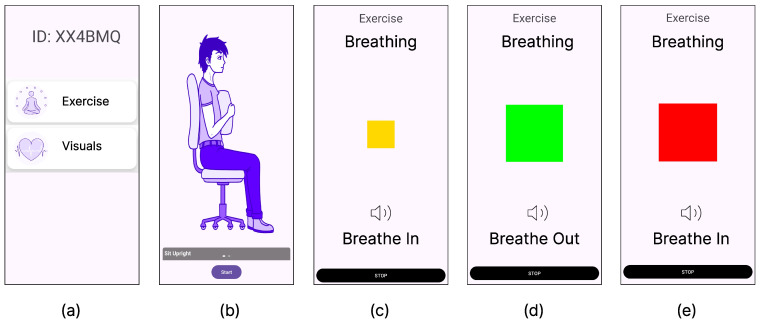
Screens of the Amiga mobile application: (**a**) main menu; (**b**) instructions for holding the cushion; (**c**) start of the breathing exercise (yellow); (**d**) phase maintained for ≥3 s (green); (**e**) phase maintained for <3 s (red).

As seen in Figure 3a the application comprises two primary functionalities and provides instructions on how to hold the cushion as seen in Figure 3b. The breathing exercise, which is shown by Figure 3c–e, guides users through the breathing exercise using synchronized voice guidance, textual instructions, and an animated visual cue that expands and contracts to represent the target breathing pattern. When the participant deviates from the prescribed breathing rhythm, the animation changes from green to red, providing immediate visual feedback to facilitate resynchronization with the exercise protocol. The second feature provides a real-time dashboard for continuous visualization of the measured respiration rate.

### 2.2. System Architecture

**Figure 4:**
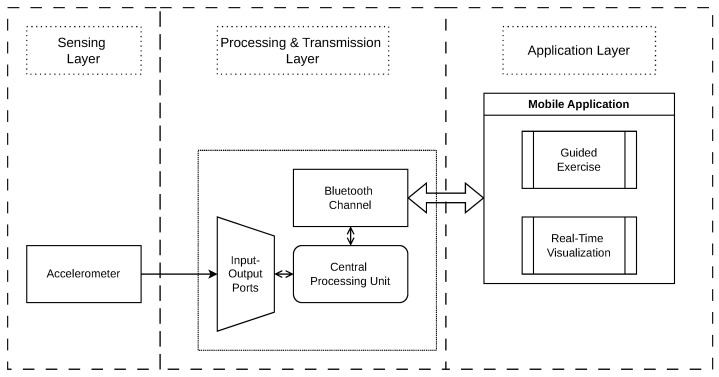
Functional Architecture of the Amiga System. The diagram illustrates the modular flow of information: the Sensing Layer captures abdomen movements through textile-integrated components; the Processing and Transmission Layer manages signal acquisition and wireless encapsulation; the Application Layer provides real-time biofeedback and session management to the user via a mobile interface.

The system is based on a modular architecture designed to support real-time physiological data acquisition and biofeedback delivery. As illustrated in Figure 4, the architecture is structured into three functional layers, each responsible for sensing, data communication and processing, and user interaction.

(a) **Sensing Layer:** Composed of accelerometer for respiration phase detection and respiration rate measurement. To preserve signal integrity during physical interaction with the cushion, the sensing element is mechanically decoupled from the main casing.
(b) **Processing and Transmission Layer:** Signals acquired from the sensing module are conditioned and processed by an onboard low-power microcontroller. The processed data are subsequently encapsulated and transmitted to the companion mobile application via the Bluetooth Low Energy (BLE) protocol.
(c) **Application Layer:** The mobile application serves as the primary user interface, managing Bluetooth connectivity, real-time data visualization, and guided breathing exercises. Incoming physiological data are processed to synchronize visual and auditory breathing cues with the user’s respiratory activity, thereby providing an intuitive and responsive interaction experience.

### 2.3. System Validation

System validation was conducted to verify that the Amiga cushion satisfies its intended functional requirements and operates reliably under defined conditions. The validation protocol and corresponding acceptance criteria were established a priori and executed by the device development team.

As with any accelerometer-based respiration monitoring systems, the cushion is inherently susceptible to motion artifacts. To mitigate this effect, validation experiments were performed under controlled stationary conditions. During testing, participants (developers) were seated in a chair with back support to ensure a stable posture and a consistent measurement environment.

Respiration rate was estimated during both spontaneous and paced breathing tasks and compared against a reference derived from manual counting of visible respiratory cycles under controlled conditions. An exploratory threshold was defined a priori, requiring at least five consecutive breathing phases with a duration of 3 ± 1 s per phase, corresponding to a target rate of 20 phases per minute sustained over the exercise period.

Manual observation is commonly used as a pragmatic benchmark in feasibility studies of abdominal accelerometer-based respiratory monitoring, where the reference RR is approximated through direct visual counting across different breathing patterns, including slow, normal, and rapid respiration [19]. The objective of this validation was not to establish clinical-grade accuracy, but to evaluate the capability of the sensing system and signal processing pipeline to distinguish between breathing modalities and to assess adherence to prescribed breathing rates in healthy participants. This approach is supported by prior studies demonstrating that accelerometer-based methods achieve performance comparable to established reference modalities, including respiratory belts, spirometers, and chest-mounted inertial sensors, with mean differences typically below one breath per minute [20,21,22].

Given this evidence and the focus on user interaction rather than clinical decision-making, the use of visually counted breaths under paced conditions provides a proportionate and methodologically sound reference, while minimizing participant burden and avoiding additional instrumentation.

### 2.4. Data Analysis

#### 2.4.1. Quality Assessment of Participants

If participants had to successfully complete the guided breathing exercise with strict adherence to the breathing exercises, they would have to achieve a pace of 20 Ph/min with a duration accuracy of 3 s for each inhalation or exhalation phase. The breathing exercise has a total duration of 2 min which should result in 40 phases. Participants were considered to have met the minimum acceptable limit to be evaluated if they completed at least 20 of the 40 phases, corresponding to 50% of the full breathing exercise.

#### 2.4.2. Pace Synchronization Accuracy of the Participants


(1) $$\mathrm{PSA}\;(\%)=\frac{\mathrm{Ph}(n)}{40}\times 100$$

As shown in Equation (1), Pace synchronization accuracy can be described as the degree to which participants were able to match the intended respiration pace throughout the task, reflecting their ability to follow the prescribed pace without substantial deviation. In general, pacing synchronization accuracy is often interpreted by comparing the target pace of 40 with the actual performed pace Ph(n), where smaller deviations indicate better control and adherence.

#### 2.4.3. Range of Tolerance for Duration Accuracy

The mean of the total Phases (${\bar{x}}_{\mathrm{Ph}}$) and The standard deviation of the total Phases ($\sigma_{\mathrm{Ph}}$) summarize the average duration and dispersion of all combined respiration intervals, where the mean absolute error of the phase (${\mathrm{MAE}}_{\mathrm{Ph}}$) measures the average absolute deviation of individual durations from the mean duration of the participant, reflecting the regularity of respiration. A tolerance range of ±0.5 s or ±1.0 s for a target phase duration of 3 s can be selected based on the variability measures from the standard deviation ($\sigma_{\mathrm{Ph}}$) and mean absolute error (${\mathrm{MAE}}_{\mathrm{Ph}}$) as it accommodates normal physiological fluctuations while still maintaining sufficient sensitivity to identify meaningful deviations from the intended respiration pace.

Ph (n) under ±0.5 s and ±1.0 s tolerances indicates the number of phases successfully synchronized to the 3 s target phase duration within respective tolerance windows. Accuracy (Acc, %) expresses these as percentages of total detected breaths. Higher duration accuracy values indicate greater consistency and adherence to the target phase duration, whereas lower values suggest increased variability or deviation from the intended phase duration. Comparing the two tolerance thresholds provides insight into the sensitivity of the assessment, where the ±0.5 s criterion reflects stricter temporal precision, and the ±1.0 s criterion captures broader physiological variability that can still be considered acceptable during controlled breathing exercises.

#### 2.4.4. Phase Duration Accuracy of the Participants


(2) $$\mathrm{PDA}\;(\%)=\frac{\mathrm{Ph}(n)\;\pm 1\;s}{40}\times 100$$

As shown in Equation (2), phase duration accuracy refers to how closely participants matched the intended length of each respiratory phase during the guided protocol. The threshold of ±1s was selected based on the (${\mathrm{MAE}}_{\mathrm{Ph}}$) for the cohort. Accurate phase timing indicates that participants were able to maintain the prescribed respiratory structure with minimal deviation from the target duration, which is important because smaller timing errors generally reflect better temporal control and stronger adherence to the exercise pacing requirement.

## 3. Research Protocol

### 3.1. Ethics Approval

Since the usability test involved human participants, ethical approval was obtained from the Research Ethics Committee of the Universidad de Diseño, Innovación y Tecnología. This committee reports to the Vice-Rector for Research and is appointed by the Rector of the University. Its mandate is to evaluate and approve research protocols in compliance with fundamental ethical and scientific principles, align research activities with international standards in bioethics and scientific integrity, ensure the protection of participants, and provide oversight and ethical certification for researchers. This usability study was approved under the identification number CEI-UDIT-2025-09-004, on 23 September 2025.

### 3.2. Participants

The usability study involved 20 participants. This sample size was chosen according to established usability research practice. Hwang and Salvendy proposed that optimal sample sizes of 10±2 participants are sufficient to reach an 80% problem discovery rate in usability evaluations [23], whereas Faulkner demonstrated that increasing the sample size beyond five users to approximately 20 participants substantially improves the breadth of usability problem detection [24]. A sample of 20 participants provided sufficient statistical power to identify interaction problems while remaining feasible within time and resource constraints. Participants which were from different departments of the Universidad de Diseño, Innovación y Tecnología (UDIT) voluntarily participated and had no prior involvement in the project. The study focused on healthy adult individuals, with the following inclusion and exclusion criteria applied to ensure data consistency:

(a) **Inclusion Criteria:**
  (i) Adults aged 18–65 years.
  (ii) Balanced gender representation.
  (iii) Healthy individuals without acute or chronic respiratory conditions.
  (iv) Normal resting vital signs.
  (v) Ability to follow verbal and written instructions.
(b) **Exclusion Criteria:**
  (i) Individuals under 18 years of age or older than 65 years of age.
  (ii) Pregnant women.
  (iii) Participants with respiratory or cardiovascular diseases, whether temporary or chronic.
  (iv) Current smokers.
  (v) Individuals with obesity.

### 3.3. Methodology

To evaluate the usability of the system, a mixed-methods approach was used, combining structured interviews, direct observation, and validated usability questionnaires. Participants were required to perform a guided breathing exercise using the cushion and the companion mobile application under controlled conditions. The protocol was structured as follows:

(a) **Introduction:**
  (i) Welcome the participant.
  (ii) Explain the purpose of the usability test.
  (iii) Explain the confidentiality arrangements for participant data.
(b) **Structured Interview:**
  (i) Collection of demographic data.
  (ii) Assessment of previous technology experience.
  (iii) Exploration of expectations about system use.
(c) **System Use:**
  (i) Delivery of task instructions.
  (ii) Direct observation of the interaction of the participants.
(d) **Questionnaire:**
  (i) Completion of the usability questionnaire.
(e) **Closing:**
  (i) Debriefing and acknowledgment of participation.

### 3.4. Acquiring Demographic Data

A structured interview was conducted prior to system use to characterize the participant sample in terms of digital literacy, technology experience, and prior exposure to health monitoring devices. This information was used to assess how factors such as age and gender might influence technology adaptability and to anticipate potential usability challenges. The interview included questions for the following:

(Qd1) Age.
(Qd2) Gender.
(Qd3) Experience in using technology, excluding messaging and social media applications.
(Qd4) Prior use of health monitoring devices, such as smartwatches or fitness trackers.
(Qd5) Ease of learning to use new apps or devices.
(Qd6) Expectations regarding the use of the system.
(Qd7) Anticipated difficulties when using the system.

### 3.5. Testing the Usability of the System

The total duration of the test varied depending on the participant’s technical proficiency, ranging from 10 to 15 min, followed by a 5-min break to reset the system for the next participant. The usability test was conducted in a temperature-controlled classroom environment, with only members of the research team present.

Participants were evaluated individually to prevent observation bias and to ensure that each participant interacted with the system without prior exposure or familiarity. No prior training or practice sessions were provided. Instructions were standardized and delivered using a scripted protocol.

The test comprised two sets of tasks: those performed by the research team to set up the session and those performed by the participant.

1. **Tasks performed by the research team:**
  Task 1: Power on the system.
  Task 2: Select the cushion on the mobile application.
  Task 3: Generate a random anonymous ID for the participant.

Participants were seated in a chair with backrest near a desk. with the cushion placed faced-up in front of them and the mobile application was opened on the menu page displaying the generated ID. The menu offered two options: begin the guided breathing exercise or view the real-time data. During the breathing exercise, participants were required to follow a structured respiration pattern delivered through animated and audio-visual cues. The exercise lasted 2 min, with alternating 3 s inhalation and 3 s exhalation intervals. The cushion detected diaphragmatic movement and relayed this information to the application. Correct timing was indicated by a green animation, whereas deviation below the 3 s threshold triggered a red indicator. Participants were permitted to cancel the exercise at any time if they felt discomfort or dizziness. Upon completion, the participants selected the real-time data option to view the respiration rate.

2. **Tasks performed by participants:**
  Task 1: Hold the cushion in the indicated position.
  Task 2: Initiate the breathing exercise via the mobile application.
  Task 3: Read the on-screen instructions provided by the application.
  Task 4: Perform the guided breathing exercise.
  Task 5: View the real-time data.

### 3.6. Assessing the Usability of the System

Participants completed a usability questionnaire immediately after performing the guided breathing exercise. The questionnaire comprised two parts: closed-ended and open-ended questions. Closed-ended questions assessed system usability using a five-level Likert scale (1 = Strongly Disagree, 5 = Strongly Agree), whereas open-ended questions captured personal opinions and qualitative feedback. Completion of the closed-ended questions was mandatory, whereas the open-ended section was optional.

The closed-ended questions were adapted from the SUS and modified to address the specific components of the prototype, namely the cushion and the mobile application. Additional elements were included to capture aspects not covered by the standard SUS, such as the perceived intuitiveness of the cushion relative to the application and overall satisfaction of the system. The positive and negative statement structure of the SUS was preserved to enable standard dimensionless SUS scoring.

(a) **Closed-Ended Questions:**
  (Q1) I think that I would like to use this system frequently.
  (Q2) I found the system unnecessarily complex.
  (Q3) I thought the system was easy to use.
  (Q4) I think I would need the support of a technical person to use this system.
  (Q5) I found that the system’s various functions were well integrated.
  (Q6) I thought there was too much inconsistency in this system.
  (Q7) I would imagine that most people would learn to use this system very quickly.
  (Q8) I found the system very cumbersome to use.
  (Q9) I felt very confident using the system.
  (Q10) I needed to learn a lot of things before I could get going with this system.
  (Q11) The instructions for using the system were awkward and difficult to understand.
  (Q12) The application interface was intuitive.
  (Q13) I needed help holding the cushion.
  (Q14) Overall, I am satisfied with my experience using the system.
(b) **Open-Ended Questions:**
  (Qo1) Was there anything particularly difficult or confusing?
  (Qo2) How did you feel holding the cushion?
  (Qo3) What suggestions for improvement would you offer?
  (Qo4) What is your overall personal opinion of the system?

## 4. Results

In this section, we present the findings from the usability evaluation of the developed system across four main areas. First, the demographic characteristics of the participants are summarized to provide context for the study sample. User performance results from participants are reported to assess the ability of the participants to complete the assigned tasks. This is followed by the System Usability Scale (SUS) results, which quantify perceived usability. Finally, responses to open-ended questions are presented to capture subjective experiences of the participants, including perceived strengths, limitations, and suggestions for improvement.

### 4.1. Demographic Profile of Participants

The 20 participants were distributed into four age groups, ranging from 18–30, 31–45, 46–60, and 60–65 years. Female participants represented the majority with a 3:2 ratio relative to male participants, whereas one participant declined to specify their gender.

**Figure 5:**
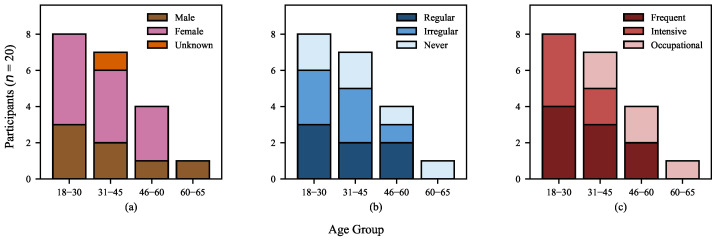
Distribution of participants (*n* = 20) by age group shown as counts: (**a**) gender; (**b**) use of smart wearables for health monitoring; (**c**) use of technology for daily tasks.

As shown in Figure 5a, the youngest age group (18–30), comprising 3 males and 5 females, showed the highest level of interest in participation. A downward trend in participation was observed with increasing age, suggesting that younger individuals may be more motivated to engage with novel technology.

Figure 5b shows the proportion of participants who had previously used smart wearable devices for health monitoring, such as smartwatches, fitness bands, chest-strap heart rate monitors or smart rings. Approximately 65% of the participants reported having used such devices regularly or irregularly, while 35% had no prior experience with this type of technology. This suggests that most of the participants were familiar with health monitoring devices at the beginning of the study, which may have influenced their expectations and interactions with the Amiga system.

Figure 5c illustrates the self-reported technology experience of participants, excluding social media and messaging applications. Participants in the 18–30 age group reported frequent use of technology for personal tasks (e.g., managing their household finances) and intensive use for advanced functions (e.g., playing online games). Participants in older age groups reported primarily occupational use related to work and occasional personal use, indicating a lower overall digital engagement.

An overwhelming majority of the participants reported confidence in their ability to learn and use new applications or devices. Most indicated that their motivation to participate stemmed from curiosity and interest in the system and expressed confidence that they would be able to interact with it without significant difficulty.

### 4.2. Evaluation of the Guided Breathing Exercise

#### 4.2.1. Quality of Participant Performance

**Figure 6:**
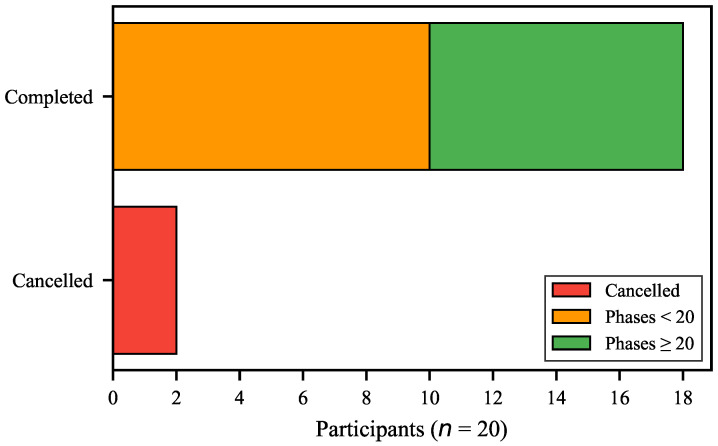
Participants (*n* = 20) distribution and exercise completion rates shown as counts: participants who completed the breathing exercise and those who canceled it by pressing the “STOP” button; participants who completed the exercise were further screened; participants who canceled and have less than 20 breathing phases were excluded from the analysis.

As shown in Figure 6, 90% (18/20) of participants completed the full 2-min exercise, while 10% (2/20) terminated it prematurely. Early terminations were attributed to boredom rather than task difficulty and occurred before completion of the session. Among participants who completed the exercise, a subset did not maintain the pace synchronization with the prescribed respiration rhythm. Additionally, the system limited phase detection (inhalation and exhalation) to a maximum of 20 phases per minute.

On average, only a subset of participants maintained a rate of 20 phases per minute throughout the full duration. Based on the predefined quality criteria, 40% of participants (8/20) met the required performance threshold.

**Figure 7:**
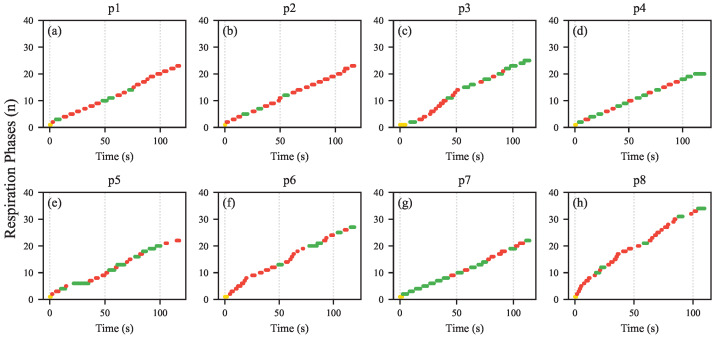
The y-axis represents the respiratory phase number, while the x-axis represents the total exercise duration (s) for each participant (n=8). The graph illustrates the respiratory phase durations for all participants. Yellow indicates the start of the exercise, red indicates phases that did not meet the prescribed duration, and green indicates phases with durations of 3 s or longer.

Figure 7 presents a visual representation of the guided breathing exercise of the 8 participants who met the quality criteria, where each inhalation or exhalation taken by the participants is represented as the respiration phase in relation to the total duration of the exercise in seconds, displayed as a color-coded interval. The initial respiratory phase is marked in yellow to indicate the beginning of exercise that begins with an inhalation phase. Subsequent respiratory phases are marked green if their duration exceeded 3 s, indicating correct execution, or red if their duration fell below 3 s, indicating a deviation from the target pattern. Nevertheless, participants maintained a general pace according to their ability, where participant p4 is seen to have a linear relationship between phase vs time, participant p8 has the most number of phases and the closest to the required pace of 40 phases, but is not maintain the required duration for the respiratory phases.

#### 4.2.2. Synchronization of the Phases and Duration

Almost all the participants were unable to adhere to the set pace and the duration as seen in Table 1 with the phase count. Participants were unable to maintain the duration of the respiration phase of exactly 3 s (Ph ≈ 3 s) most of the time, but participant p7 was able to maintain it only 5 times. In general, the participants had most of their respiration phases below the threshold of 3 s (Ph < 3 s) and a satisfactory level above 3 s (Ph > 3 s). Participant p8 was able to achieve 34 phases reflecting in the RR of 8, which is higher than the average of the rest of the participants.

#### 4.2.3. Accuracy of the Pace Synchronization and Phase Duration with Tolerance

**Figure 8:**
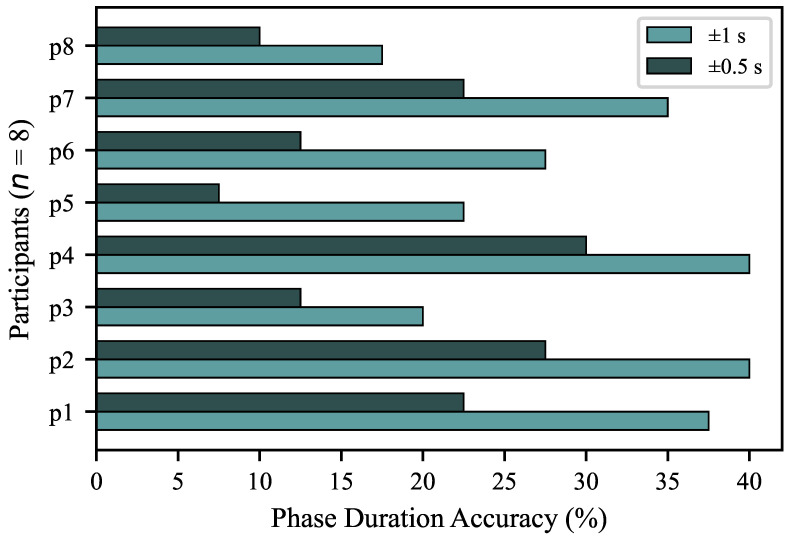
Phase duration accuracy of participants (*n* = 8) for 3 s target phases under ±0.5 s and ±1.0 s tolerances.

Table 2 presents the pace synchronization and phase duration performance of each participant during the breathing exercise. As seen in Figure 8. It can be said that participant p4 achieved the highest overall duration accuracy of 30% with a tolerance of ±0.5 s and 40% with a tolerance of ±1 s, followed by participant p2. Regarding the accuracy of pace synchronization, participant p8 achieved 85% followed by participant p6 who achieved 67.5%.

### 4.3. Evaluation of the Usability

Upon completion of the breathing exercise, participants responded to the 14-item usability questionnaire. Table 3 presents the percentage distribution of responses for each item using a five-level Likert scale, where 1 = Strongly Disagree (SD), 2 = Disagree (D), 3 = Neutral (N), 4 = Agree (A), and 5 = Strongly Agree (SA).

The response distribution reveals several notable patterns. Strong positive agreement was observed for Q3 (ease of use), Q9 (confidence), and Q14 (overall satisfaction), where all participants reported agreement or strong agreement. Conversely, strong disagreement was recorded for Q2 (unnecessary complexity), Q6 (inconsistency), Q8 (cumbersomeness), and Q11 (difficulty of instructions), all of which are negatively worded items in the SUS structure. The disagreement with negative statements is consistent with a positive perception of usability and contributes positively to the overall SUS score. The SUS score was computed using only the first 10 questions, in accordance with the standard scoring method.

**Figure 9:**
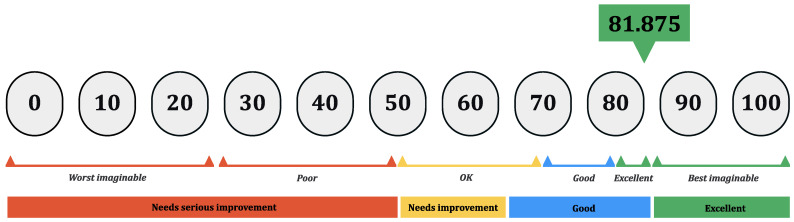
Visual representation of the mean SUS score obtained from the responses to the usability evaluations of the participants (*n* = 20): A mean SUS score of 81.875 was obtained with standard deviation (σ = 11.8) corresponding to the rating of the category ‘Excellent’.

The quantitative analysis of the usability assessment yielded a mean SUS score of 81.875 with standard deviation (σ = 11.8), as illustrated in Figure 9. According to established empirical benchmarks [25], this score places the system in the Excellent category, well above the industry average of 68. This result is further reinforced by the unanimous positive responses to confidence (Q9) and overall satisfaction (Q14), with 100% of participants reporting a positive response. Notably, Q5 (integration of system functions) received the highest proportion of strong agreement (65%), suggesting that participants perceived the cushion and the mobile application as a coherent and well-integrated system. These results are consistent with a coherent integration of the cushion and the application from the user’s perspective.

#### Relationship Between Usability Ratings and Participant Performance

Spearman rank-order correlation was performed to investigate the association between SUS scores and phase duration accuracy at ±1 s of the participants selected from the quality criteria. The results indicated a weak negative and non-significant correlation between the two measures, the correlation coefficient ($r_{s}=-0.13$) and the *p*-value (p=0.756), suggesting that subjective usability ratings were not significantly associated with duration accuracy performance.

### 4.4. Open-Ended Responses

The open-ended responses highlighted several areas for improvement across the cushion’s physical design, the mobile application, and the breathing exercise. In general, participants did not report significant difficulty or confusion when interacting with the system, and most described holding the cushion as comfortable.

#### 4.4.1. Smart Cushion

Participants suggested several design modifications to improve comfort and intuitiveness, including deeper hand pockets, clearer orientation cues, and a more huggable form factor. Some proposed alternative designs that would encourage a fuller, more embracing posture, whereas a few reported mild discomfort with hand placement on the cushion surface. These observations suggest that the overall form factor was well received, and refinements to the physical interface could further improve the naturalness of interaction.

#### 4.4.2. Mobile Application

The most frequently reported issue concerned the clarity of the visual instructions. Several participants misunderstood the on-screen guidance on how to hold and position the cushion, indicating that the current instructions were insufficient for first-time users. Participants also requested in-application feedback to detect and alert when the cushion is not held correctly, as well as more detailed explanations of the metrics displayed on the dashboard. These findings point to the need for improved onboarding and contextual guidance within the application.

#### 4.4.3. The Breathing Exercise

Participants suggested that the ability to customize exercise parameters, such as inhalation and exhalation durations, would enable a more personalized experience. The voice prompts reminding users to inhale or exhale were perceived as repetitive or intrusive by some participants, who recommended reducing their frequency. Notably, one participant reported increased anxiety when attempting to strictly adhere to the prescribed respiration pattern, highlighting the importance of offering more flexible and mindfulness-based exercise alternatives in future iterations.

Despite these observations, participants responded positively to the overall concept, perceiving the system as useful, comfortable, and innovative. Several emphasized the portability of the cushion and its potential for guided breathing exercises. Most of the participants described their experience as satisfactory and expressed interest in future developments of the technology. In general, the qualitative feedback was overwhelmingly positive.

## 5. Discussion

This study evaluated the usability of a cushion-based smart textile system for guided breathing exercises with a population of 20 participants. Overall, the findings suggest that the system was feasible and positively received, while also revealing important opportunities to improve adherence, guidance, and first-use experience.

The predominantly young female adults and technologically confident participants likely shaped the usability findings as prior experience with smart devices and health-monitoring apps may have lowered the learning curve and inflated perceived ease of use, independent of the system’s inherent design quality. This suggests that usability scores may not generalize to less technology familiar populations such as older adults or participants with limited digital literacy.

The breathing exercise results suggest that task completion alone does not guarantee sustained engagement or compliance with the instructed rhythm. Although most participants completed the exercise, some disengaged midway, reporting boredom, which indicates that repetitive breathing tasks may reduce attention and motivation over time. Several participants did not adhere to the prescribed pace and duration, instead reverting to a self-selected breathing rhythm that may have felt more natural or comfortable. This pattern suggests that externally imposed pacing can be difficult to maintain, especially when the task requires sustained concentration and behavioral synchronization. The findings indicate that while the exercise was feasible to complete, strict adherence to the target protocol was limited, highlighting a need for more engaging guidance strategies and perhaps greater flexibility in pacing design.

The high acceptability of the system suggests that the guided breathing exercise provided adequate support for first-time users, as most participants were able to complete the task with minimal assistance. The few participants who did not meet the quality criteria appear to have struggled more with following the breathing pattern than with using the system itself, which suggests that the main limitation was not technical robustness but adherence to the exercise protocol. The favorable usability ratings may have been influenced not only by the mobile interface but also by the cushion’s comfort, appearance, and interactive design, all of which likely contributed to a positive user experience. The very weak negative association between usability and duration accuracy further suggests that higher perceived usability did not necessarily translate into stricter compliance with the prescribed breathing timing.

Comfort-related observations were obtained from participant’s responses to the open-ended questions in the questionnaire and therefore reflect subjective user perceptions rather than quantitative comfort measurements. Participants generally reported a positive experience after a brief familiarization period, suggesting that the cushion-based format was intuitive and approachable for first-time use. The main usability issues concerned physical refinement rather than conceptual acceptance, particularly hand placement, pocket size, and the clarity of the initial instructions. This indicates that the system’s core interaction was well received, but the first-use support and ergonomic details could be improved to reduce minor sources of friction. The report of increased anxiety in one participant also highlights that rigid adherence to a prescribed breathing rhythm may not suit all participants.

The cushion was selected as the primary interaction interface based on the concept of comfort [12], suggesting that physical contact with a familiar soft item can produce a calming effect. Unlike traditional wrist-worn wearables, the Amiga system leverages this natural affordance to lower the barrier to engagement with breathing-based exercises. The participants who met the quality criteria demonstrate that the system provides sufficient guidance for the first time where the performance of the participants totally depended on the ability of the participants to follow paced breathing. Although participants were not able to accurately follow the pace and duration, the observed behavior may reflect an individually consistent but unprescribed breathing pattern, rather than poor performance. Consequently, pace synchronization accuracy and duration accuracy should not be interpreted as the sole indicator of respiration quality or task execution.

On average, participants achieved only moderate pace synchronization and low duration accuracy, indicating limited adherence to the target breathing protocol. However, the fact that one participant closely matched the prescribed pace suggests that the observed variability cannot be attributed solely to device limitations. Instead, performance likely reflected a combination of system-related factors and participant-specific differences, such as responsiveness to feedback, interpretation of the instructions, and individual pacing ability. To our knowledge, no prior studies have reported participant-level adherence percentages or pace synchronization accuracy for wearable or smartphone-guided breathing exercises, which limits direct comparison with existing approaches.

This study demonstrates the feasibility of a non-intrusive monitoring approach for guided breathing exercises rather than providing a full validation of respiration sensing for clinical use. The system was developed to reduce the need for body-worn hardware (for example, smart shirts, wristbands, or adhesive patches and straps), which can cause discomfort or limit movement [26,27,28]. By avoiding sensors that must be strapped or attached to the body, the approach prioritizes user comfort and unobtrusive integration into daily activities, consistent with principles of ubiquitous computing and user-centred design. however, validation with instrumented reference sensors is required to quantify its accuracy which is left for the future work.

Future research should prioritize longitudinal evaluations under ecologically valid, unsupervised conditions to capture temporal dynamics in user behavior, and perception. Expanding cohort size and diversity will be essential to ensure external validity and generalizability. In addition, several key questions remain unresolved, including the extent to which variations in the form factor of the cushion and the feedback modalities influence comfort, engagement, and sustained use over time. Finally, further work is needed to explore a broader spectrum of breathing and mindfulness-based interventions, alongside the development of adaptive, user-specific feedback mechanisms that can accommodate inter-individual variability in preferences and physiological responses.

## 6. Conclusions

This study demonstrates the feasibility of integrating sensors into everyday textile objects to support self-guided breathing exercises, which could in the future offer a comfortable and familiar alternative to clinical or professional-led interventions. The cushion-based smart textile system was well received by users, with most participants completing the guided exercise independently and reporting high confidence and satisfaction, supporting its potential as an accessible tool for relaxation-focused, home-based breathing exercises. This work provides feasibility evidence that smart textiles can lower the barrier to engagement by embedding physiological sensing within a comfortable, approachable form factor. Future work should validate the system in clinical settings, refine on-screen guidance, evaluate long-term usability in unsupervised real-world settings with larger and more diverse populations, and explore adaptive, mindfulness-based exercise variants tailored to individual needs.

## 7. Patents

We are currently in the preliminary analysis phase of the patent for this research work.

## Figures and Tables

**Table 1:** Overview of the breathing exercises per participant. Ph (n): number of phases; RR (n): number of respiration cycles per minute; Ph < 3 s (n): phases shorter than 3 s; Ph ≈ 3 s (n): phases of approximately 3 s; Ph > 3 s (n): phases longer than 3 s; ${\bar{x}}_{\mathrm{Ph}}$ (s): mean duration of all phases (inhale and exhale); $\sigma_{\mathrm{Ph}}$ (s): standard deviation of all phase durations; ${\mathrm{MAE}}_{\mathrm{Ph}}$ (s): mean absolute error of all phase duration; ${\bar{x}}_{G}\pm \sigma_{G}$: group mean ± standard deviation.

Id	Ph (n)	RR	Ph			${\bar{x}}_{\mathrm{Ph}}$ (s)	$\sigma_{\mathrm{Ph}}$ (s)	${\mathrm{MAE}}_{\mathrm{Ph}}$ (s)
			(n) < 3 s	(n) ≈ 3 s	(n) > 3 s			
p1	23	6	19	1	4	2.20	0.95	0.73
p2	23	6	20	2	3	2.22	0.93	0.71
p3	25	6	14	1	10	2.51	1.50	1.28
p4	20	5	9	3	11	3.04	1.57	0.79
p5	22	6	14	1	8	2.89	2.87	1.86
p6	27	6	22	2	5	1.90	1.47	1.08
p7	22	6	9	5	12	3.01	0.89	0.68
p8	34	8	29	1	5	1.37	1.14	0.84
${\bar{x}}_{G}$	24.5	6.12	17.0	2.0	7.25	2.39	1.42	1.0
$\pm \sigma_{G}$	4.38	0.83	6.85	1.41	3.45	0.59	0.65	0.41

**Table 2:** Phase pace synchronization accuracy and phase duration accuracy across participants and duration at tolerances of ±0.5 s and ±1.0 s. Ph (n): number of phases; sub-columns show Ph (n) and accuracy (%). ${\bar{x}}_{G}\pm \sigma_{G}$: group mean ± standard deviation.

Id	Ph (n)	Ph	±0.5s		±1s	
		Acc (%)	Ph (n)	Acc (%)	Ph (n)	Acc (%)
p1	23	57.5	9	22.5	15	37.5
p2	23	57.5	11	27.5	16	40
p3	25	62.5	5	12.5	8	20
p4	20	50	12	30	16	40
p5	22	55	3	7.5	9	22.5
p6	27	67.5	5	12.5	11	27.5
p7	22	55	9	22.5	14	35
p8	34	85	4	10	7	17.5
${\bar{x}}_{G}$	24.5	61.25	7.25	18.12	12.0	30.0
$\pm \sigma_{G}$	4.38	10.94	3.41	8.53	3.7	9.26

**Table 3:** Participants (*n* = 20) responses of the usability evaluation by percentage distribution. 1 = Strongly Disagree (SD), 2 = Disagree (D), 3 = Neutral (N), 4 = Agree (A), and 5 = Strongly Agree (SA).

Q	SD (%)	D (%)	N (%)	A (%)	SA (%)
	1	2	3	4	5
Q1	0	0	20	55	25
Q2	60	30	10	0	0
Q3	0	0	0	45	55
Q4	40	25	10	25	0
Q5	0	0	0	35	65
Q6	50	35	10	5	0
Q7	0	0	10	30	60
Q8	60	40	0	0	0
Q9	0	0	0	45	55
Q10	30	15	10	40	5
Q11	45	30	15	10	0
Q12	0	5	10	35	50
Q13	30	15	0	25	30
Q14	0	0	0	20	80

## Data Availability

Data is unavailable due to privacy and ethical restrictions.

## Abbreviations

The following abbreviations are used in this manuscript:

SUS	System Usability Scale
Ph	Respiratory Phase
RR	Respiration Rate
DASS-21	Depression Anxiety Stress Scales—21
BLE	Bluetooth Low Energy
PSA	Pace synchronization accuracy
PDA	Phase duration accuracy
